# Whole-body MRI in children: state of the art

**DOI:** 10.1259/bjro.20210087

**Published:** 2022-10-27

**Authors:** Trevor Gaunt, Paul D Humphries

**Affiliations:** 1 Radiology Department, University College London Hospitals NHS Foundation Trust, London, United Kingdom; 2 Radiology Department, Great Ormond Street Hospital for Children NHS Foundation Trust, London, United Kingdom

## Abstract

Whole-body magnetic resonance imaging (WBMRI) is an increasingly popular technique in paediatric imaging. It provides high-resolution anatomical information, with the potential for further exciting developments in acquisition of functional data with advanced MR sequences and hybrid imaging with radionuclide tracers. WBMRI demonstrates the extent of disease in a range of multisystem conditions and, in some cases, disease burden prior to the onset of clinical features. The current applications of WBMRI in children are hereby reviewed, along with suggested anatomical stations and sequence protocols for acquisition.

## Introduction

Whole-body magnetic resonance imaging (WBMRI) is a technique gaining popularity in paediatric imaging. Without the use of ionising radiation, it can provide anatomical and functional data regarding the extent of disease and, at times, prior to the onset of clinical manifestation. There are limited number of clinical studies validating the impact and precision of WBMRI in children, and the variable signal characteristics of developing bone marrow should prompt caution to avoid ascribing pathological significance to normal findings. Despite this, WBMRI is becoming increasingly attractive for a range of paediatric multisystem pathologies, to the extent that the European Society of Paediatric Radiology Oncology Taskforce recently published recommendations for its deployment.^1^ The current applications of WBMRI in children are hereby reviewed.

### Sequences and parameters

**Figure 1.**
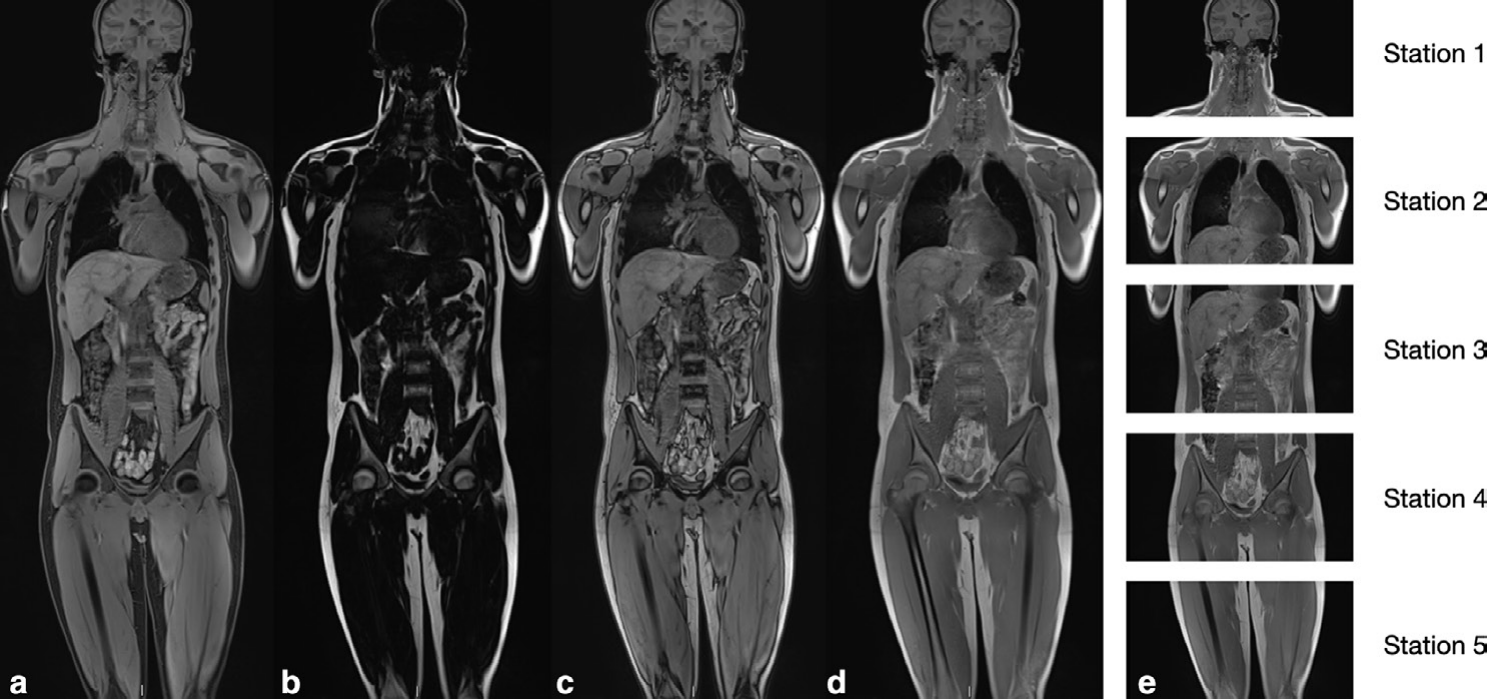
Two point Dixon T1 GRE coronal WBMRI for lymphoma; water (**A**) fat (**B**), out-of-phase (**C**) and in-phase (**D**) images. WBMRI is acquired in five stations as shown (**E**). The lower legs and feet are acquired separately if needed.

A recent systematic review of 56 studies investigating WBMRI in children showed the vast majority were performed at 1.5 T using a short tau inversion recovery (STIR) sequences, mostly in the coronal plane. STIR sequences provide reliably uniform fat suppression across a large field of view, with good contrast between pathological fluid signal intensity and suppressed fat. Other studies used complimentary *T_1_*-weighted (T1W) or diffusion-weighted imaging sequences (DWI), or both, and a minority used gadolinium contrast medium.^2^ Technical advances have led to techniques with improved signal-to-noise ratio such as T2 with spectral fat saturation and T2 Dixon, with the latter providing images with and without fat suppression. Furthermore, the generated fat-only reconstruction can replace separate T1 sequences in the investigation of marrow pathology, reducing total scanning time.^3^ The sequence parameters and anatomical acquisition stations used at our institutions are shown in Tables 1–3 and Figure 1.

**Table 1.** WBMRI protocol for Lymphoma

	Axial T2 HASTE	Axial DWI^a^/ADC	Axial T1 VIBE DIXON	Coronal T1 VIBE Dixon
Field of view (cm)	45.7 × 72.3	45.7 × 72.3	45.7 × 72.3	51.0 × 80.7
Matrix	320 × 512	320 × 513	320 × 514	192 × 727
Repetition time (ms)	878	4500	3.97	4.21
Echo time (ms)	119	58	2.39/4.77^b^	2.39/4.77^b^
Excitations	1	1	1	1
Slice width (mm)	5	5	3	5

Whole body MRI Paediatric Lymphoma protocol.

a DWI performed at B0, B50, B800.

b TE1/TE2 T1 VIBE Dixon. Axial and coronal T1 VIBE Dixon performed pre- and post-Gadolinium.

**Table 2.** WBMRI protocol for cancer predisposition syndromes

	Axial T2 TSE	Axial DWI/ADC
Field of view (cm)	41.7 × 41.7	41.7 × 41.8
Matrix	320 × 320	200 × 200
Repetition time (ms)	5320	5500
Echo time (ms)	98	61
Excitations	1	3
Slice width (mm)	4	6

**Table 3.** WBMRI protocol for chronic recurrent multifocal osteomyelitis

	Coronal STIR	Coronal T1 TSE
Field of view (cm)	37.9 × 34.3	37.9 × 34.3
Matrix	320 × 320	320 × 320
Repetition time (ms)	5193	700
Echo time (ms)	101	17
Inversion time (ms)	190	-
Excitations	1	2
Slice width (mm)	5	5

### Findings in healthy children

Bone marrow assessment in children and adolescents can prove challenging, particularly in infiltrative diseases such as lymphoma. To determine what is pathological, it is necessary to understand the variation in appearance that occurs throughout childhood and adolescence. Some physiological processes are well documented, while mimics of disease are being increasingly recognised in normal children, as more studies of normal volunteers are conducted.

Red-to-yellow marrow conversion occurs throughout childhood an into early adulthood. Conversion follows a predictable centripetal pattern along the long bones, eventually being confined to the proximal humeri, proximal femora and the axial skeleton by the third decade of life. Moreover, red marrow reconversion occurs in the opposite order and occurs at times of increased hematopoietic demand.

A complete description of marrow maturation patterns in children is beyond the scope of this review and is more comprehensively detailed elsewhere in the literature.^4^

Shabashin et al showed a symmetric and fairly consistent pattern of endosteal high T2 signal in the feet and ankles of children, with changes observed in the bone marrow of 45/402 bones (11%) and in 24/41 patients younger than 16 years (59%). These changes were most commonly in the calcaneus, talus and navicular, and typically disappeared after the age of 15 years.^5^

Avenarius et al examined the wrists of healthy children over a period of four years. Oedema-like T1 and T2 marrow changes seen on an initial scan were either no longer present or in different locations four years later suggesting changes, which might mimic disease, may be transient and resolve over time. Half the subjects showed a joint fluid pocket of more than 2 mm. Ganglion cysts were observed in 24%, with some regressing and others appearing over the 4-year period.^6^

Ordling Muller et al investigated the accuracy of MRI in detecting early bony destruction in JIA, on the basis that bony depressions at the wrist resembling erosions are frequently seen on MRI in healthy children. In a comparison of 68 children with JIA and 85 healthy controls between the ages of 5 and 15 years, they found no significant difference in the number of bony depressions between the two groups at any age.^7^

The same group demonstrated that healthy children frequently have focal, sometimes asymmetrical, non-pathological regions of restricted diffusion in their marrow, questioning whether DWI would be accurate in paediatric cancer staging. Forty-two children had areas of high DWI signal in the lumbar vertebral bodies and bony pelvis, with a tendency toward a reduction in the extent of high signal within each bone with age, but also a widespread individual variation.^8^

Finally, normal findings mimicking pathology on WBMRI are not limited to older children and the bone marrow. Clark et al showed the presence of fascial fluid was a normal finding in postpartum neonates, likely related to the birthing process of labor and not an indication of underlying pathology.^9^ Caution should therefore be exercised when interpreting these complex whole-body examinations.

### Inflammatory conditions

#### CRMO

Chronic recurrent multifocal osteomyelitis (CRMO) is an autoinflammatory condition characterised by episodes of clinical and serological systemic inflammation in the absence of autoantibodies, pathogens or antigen-specific T-cells. There have been multiple small studies into the use of WBMRI in children with CRMO which have yielded similar findings. The most frequent MRI findings are bone marrow oedema, osteitis and periosteal reaction. Most patients have multifocal bone lesions, more commonly involving the shoulder girdle, vertebrae and long bones.^10–14^

**Figure 2.**
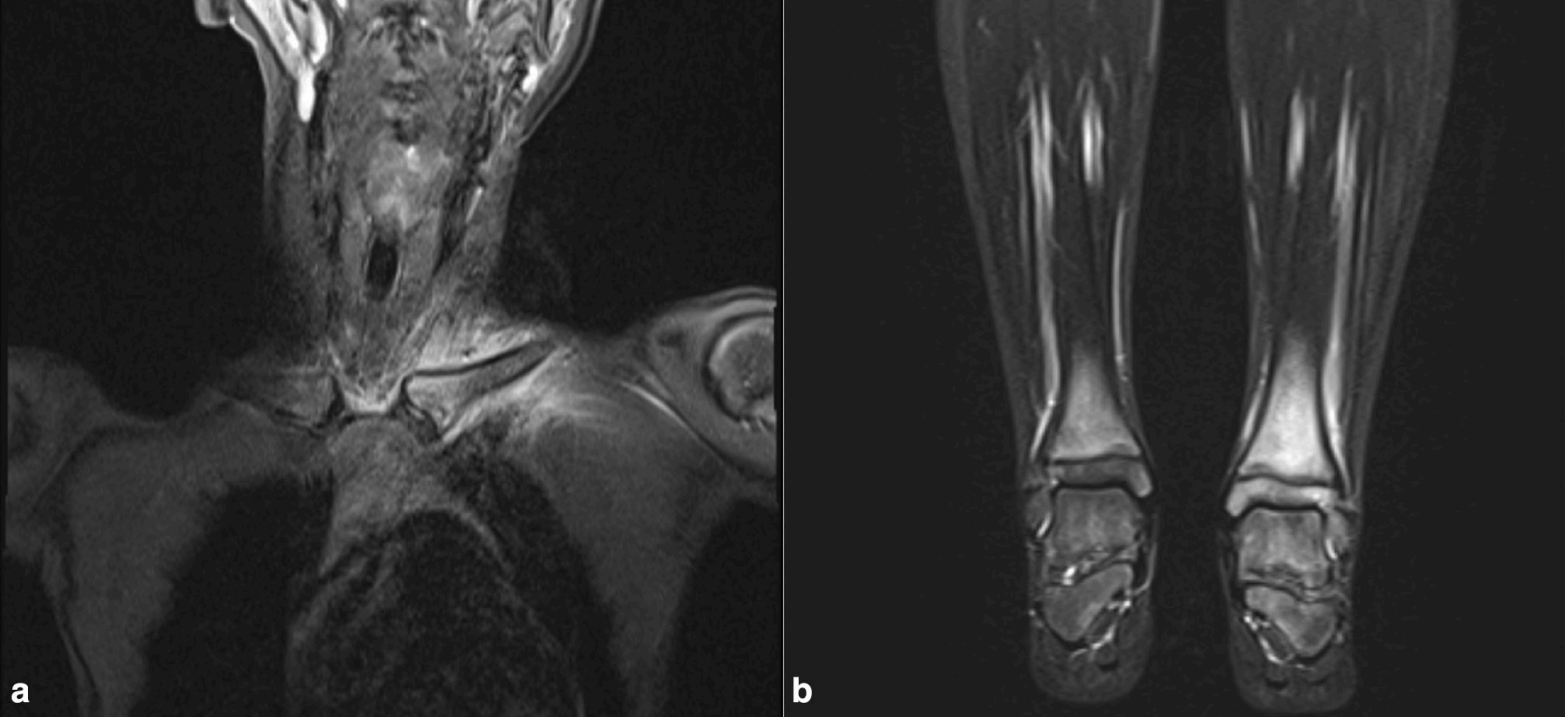
Coronal STIR sequences through the chest (**A**) and ankles (**B**) show high marrow signal, with associated periostitis and soft tissue oedema in the left clavicle and both distal tibial metaphyses.

WBMRI has been shown to be more sensitive in the detection of inflammatory bone lesions in patients with CRMO than other modalities^13,15^ (Figure 2).

Fritz et^13^ al studied T1, STIR and contrast-enhanced T1 whole-body MRI against plain radiographs, clinical findings and laboratory data in 13 children with CRMO. Coronal sequences were performed through the head, thorax, abdomen, pelvis, and limbs, with targeted axial sequences in regions of suspected pathology. Laboratory test results were negative for antinuclear antibodies, human leukocyte antigen B27, bacterial antigens and bacteremia in all children, as were white blood cell and red blood cell counts.

MRI depicted a total of 101 ill-defined oedema-like osseous lesions. The most frequent sites were the distal femur (21%,), proximal tibia (17%), and distal tibia and fibula (14% each). Seventy lesions were found in tubular bones, more frequently in the metaphysis (86%) than epiphysis (67%), diaphysis (14%) or apophysis (3%). Lesion continuity with the physis was seen in 89% and periosteal reaction in 48%. Symmetrical involvement was seen in 85% of patients, most commonly in the knees and ankles when present. MRI demonstrated multifocality in all patients. No lesions were found in the cranium, clavicle or upper limb. No periosteal reaction was seen in the vertebrae or sacrum. There were no extraskeletal abnormalities and no relationship between serum inflammatory markers and number of symptomatic anatomic sites. Compared to MRI, sensitivity for radiography was 0.13, physical examination, 0.31, and serum inflammatory markers, 0.15.

Von Kalle et al demonstrated that 52 of a study population of 53 children and adolescents with histologically or clinically confirmed CRMO demonstrated multifocal lesions on WBMRI when only 26 of 53 had presented with multifocal symptoms. This suggests that WBMRI could demonstrate lesions prior to clinical manifestations,^16^ although there is a lack of evidence to suggest that the presence of clinically occult lesions on MRI is of prognostic significance.

Notwithstanding the variability of DWI signal among the marrow of normal individuals mentioned above, Leclair et al suggested ADC values were substantially elevated in CRMO lesions at 3T in a small study of 16 patients with confirmed CRMO. By calculating the ratio of the lesion ADC value to a contralateral normal signal “reference point”, 76% showed a value increase in CRMO lesions of more than 15%.^17^

More recently, Andronikou et al suggested CRMO could be classified according to the distribution of marrow signal abnormality. The group noted children with multifocal involvement, termed “tibio-appendicular multifocal” pattern in this paper, more commonly had marrow signal abnormalities in the appendicular skeleton, whereas those, in contrast with Fritz et al above, with a “claviculo-spinal pauci-focal” pattern more commonly involved the axial skeleton. Only 5 of 37 children between 2 and 18 years of age had synchronous tibial and clavicular lesions.^18^

Hospach et al showed that reversal of symptomatic abnormal signal intensities and mild associated deformities in the spine can be achieved with treatment. In this study, of 27 CRMO patients with a total of 72 vertebral lesions, 19 complained of back pain. Seven patients with pain had spinal deformities and oedema like signal change and were treated with pamidromate. On follow-up WBRMI after 13 months, all signal abnormalities had resolved and improvement of vertebral height was seen in a total of three vertebrae in two patients.^19^ On the contrary, after achieving clinical remission in seven of eight patient’s refractory to NSAIDs, glucocorticoids and sulfasalazine, with six cycles of pamidronate, Hoffmann et al showed subclinical bone inflammation was still detectable by MRI in most patients after six months. Moreover, one patient developed radiological progression despite a marked improvement of clinical symptoms.^20^

Similarly, others have shown that new radiologically apparent lesions can occur in myriad locations with variable chronicity but may be clinically occult. WBMRI may be very sensitive for initial diagnosis and follow-up, but caution should be exercised in ascribing pathological significant to radiological features without clinical context.^21^

#### Juvenile dermatomyositis

**Figure 3.**
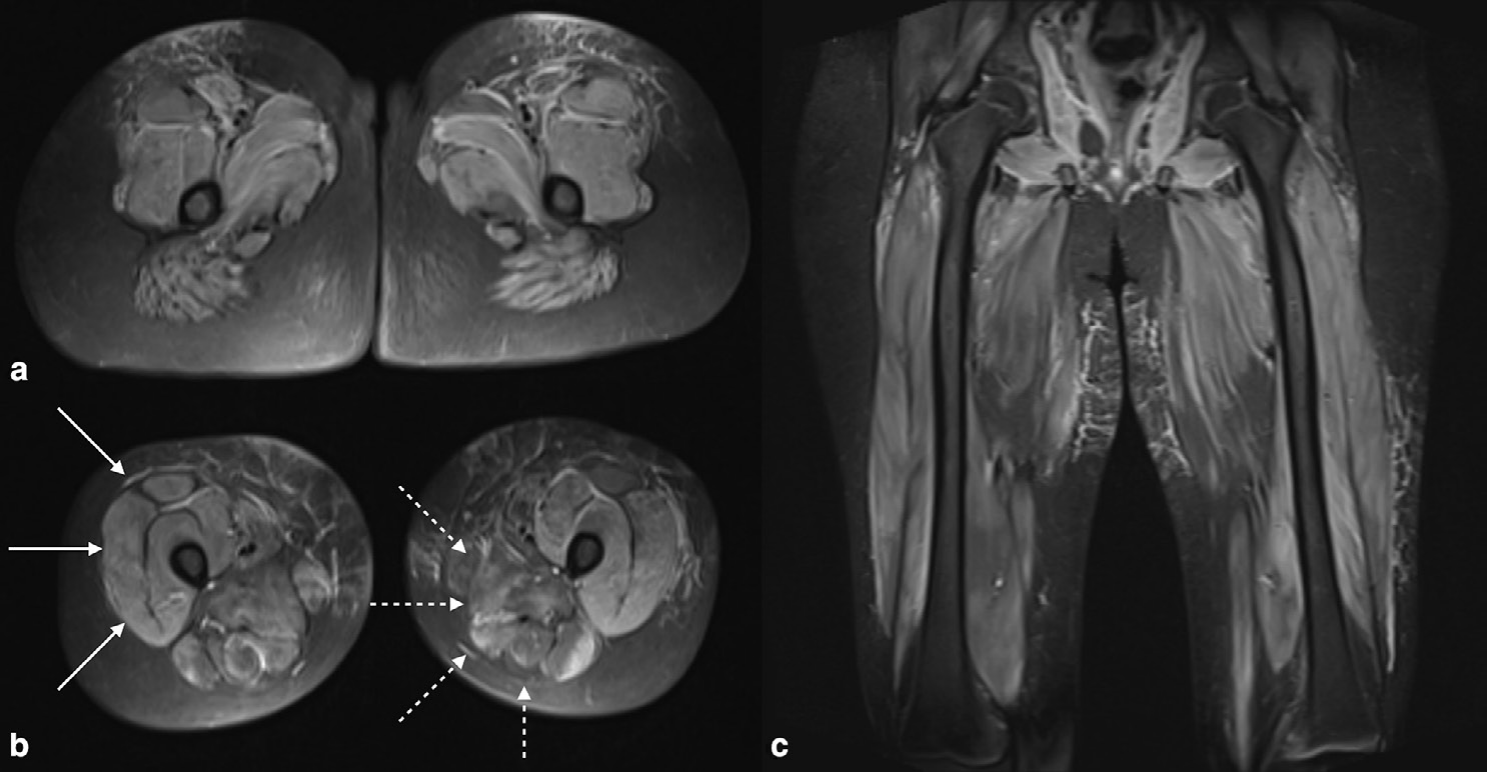
Axial and coronal STIR sequences through the thighs of a 13-year-old with juvenile dermatomyositis. High STIR signal is seen in the muscle bellies of all muscles in all compartments of the thighs. There is associated muscle atrophy in the adductor and posterior compartments bilaterally (dashed arrows in B), whereas the anterior compartments are relatively spared (solid arrows in B).

Juvenile dermatomyositis (JDM) is a cell-mediated inflammatory myopathy targeted at striated muscle with resultant atrophy, oedema, coagulation necrosis, fibrosis and calcification. WBMRI is not currently widely used in JDM, with thigh muscle imaging being most employed. However, WBMRI has been used by some to demonstrate clinically occult lesions elsewhere, potentially demonstrating the total inflammatory burden in JDM patients (Figure 3).

Abnormal muscle, subcutaneous and myofascial tissues are generally high T2 signal throughout the affected region, and calcification appears as low signal. Malattia et al performed WBMRI in 41 JDM patients and 41 control subjects and scored signal abnormalities in muscle, subcutaneous and myofascial tissues across 36 muscular groups in the extremities. WBMRI revealed clinically occult inflammatory signal in the distal legs and forearms, with 26 patients showing a patchy distribution.^22^

Similarly, Castro et al showed abnormal muscle signal in 4 of 34 dermatomyositis (JDM)/polymyositis (JPM) patients. These regions correlated with positive muscle biopsy and raised abnormal muscle enzymes. WBMRI therefore could provide additional detail to estimate total inflammatory burden, guide potential biopsy targets and monitor response to treatment.^23^

### Haematological conditions

#### Lymphoma

**Figure 4.**
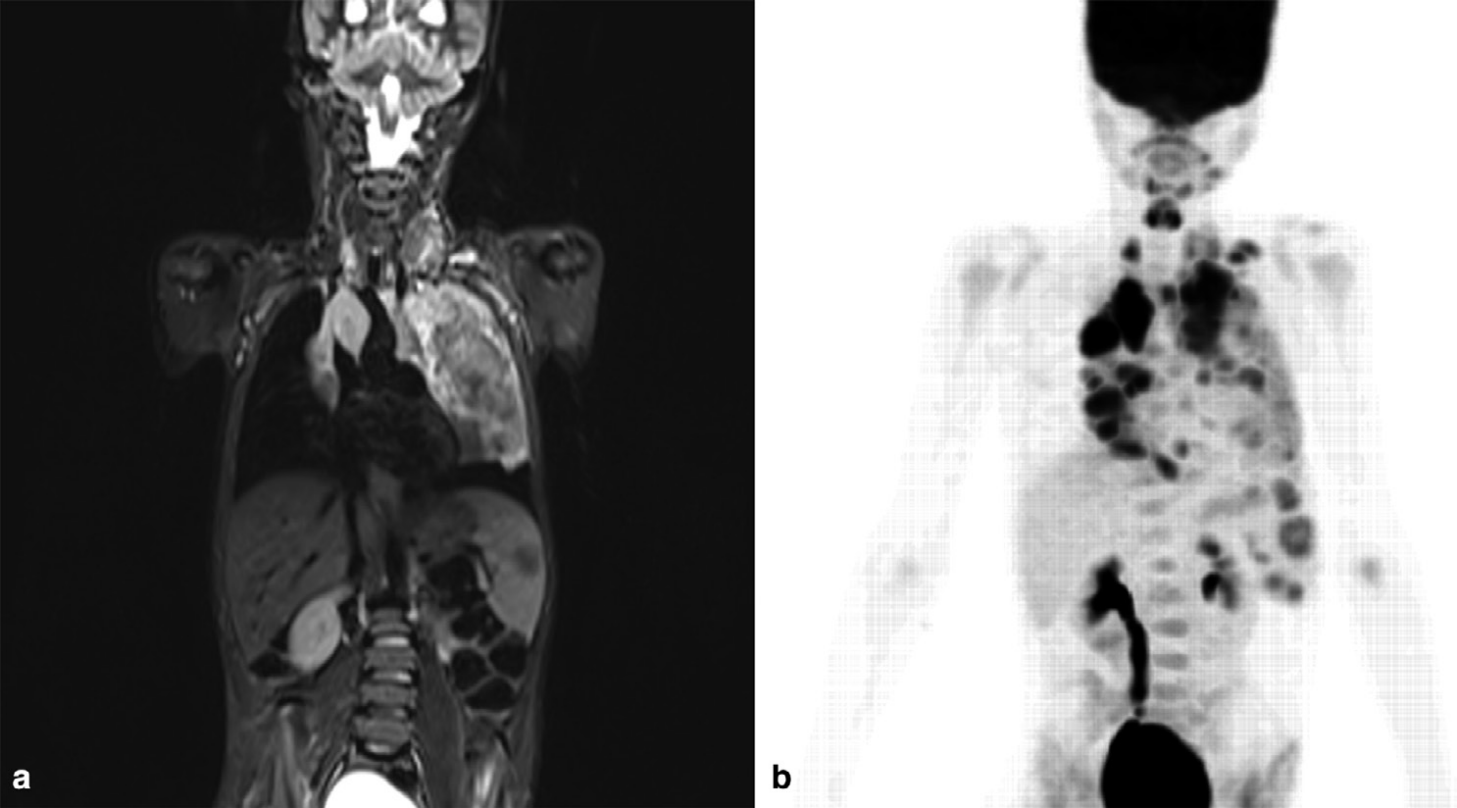
Coronal whole-body STIR MRI sequence (**A**) demonstrates gross mediastinal lymphadenopathy with left lung infiltration, and splenomegaly with a focal lymphomatous deposit in a 9-year-old boy with Hodgkin’s Lymphoma. (**B**) Whole-body maximum intensity projection image from the concomitant 18-FDG-PET/MRI examination demonstrates disease above and below the diaphragm and obstructive right hydronephrosis.

WBMIR is a reliable and sensitive tool for the staging and follow-up of Hodgkin’s Lymphoma.^24–31^ WBMRI utilising STIR sequences for staging in lymphoma has been used for at least the last decade. Kellenberger et al showed in 2004 that WBMRI STIR detected more sites of possible lymphomatous involvement at initial staging and at re-staging than did conventional imaging with CT, particularly with regards to bone marrow involvement.^24^ Furthermore, Punwani et al showed good agreement between MRI and 18-FDG-PET/CT for nodal and extranodal staging using STIR and RARE WBMRI^26^ (Figure 4).

More recently, WBMRI with DWI has been compared to 18-FDG-PET/CT for initial staging and interim response evaluation in paediatric and adolescent Hodgkin’s Lymphoma. Spijkers et al found WBMRI DWI agreed with the initial staging 18-FDG-PET/CT reference standard in 96% of 68 children with HL, with excellent interrater agreement for both intra- and extranodal staging.^27^

**Figure 5.**
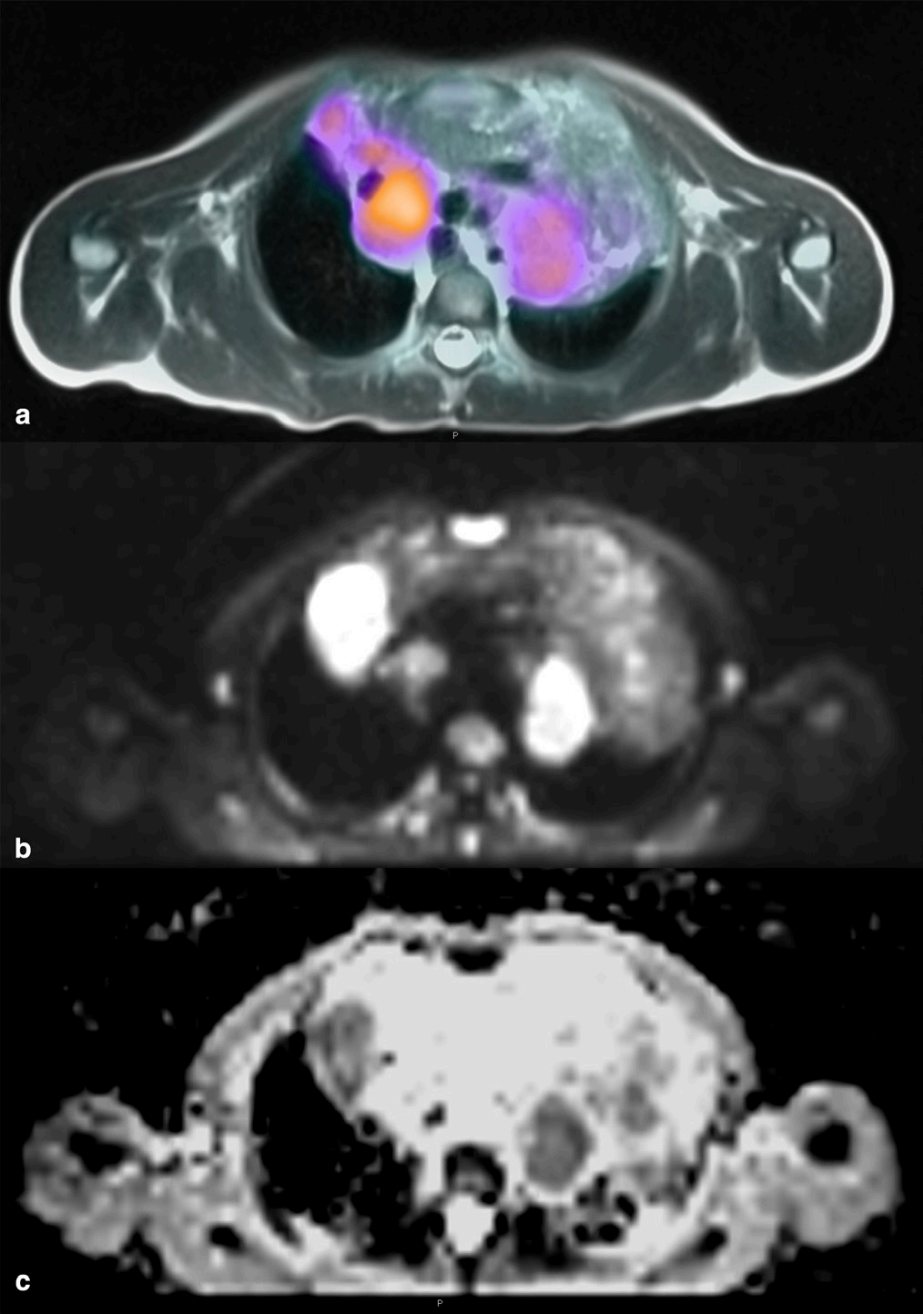
Axial fused 18-FDG-PET and T2 HASTE MRI examinations (**A**) demonstrating avid tracer uptake in a large mediastinal mass in a 9-year-old boy with Hodgkin’s Lymphoma. Axial diffusion weighted b800 image (**B**) and corresponding ADC map (**C**) demonstrating abnormal restricted diffusion in the same regions showing 18-FDG tracer avidity.

DWI and ADC have previously been shown to compliment PET for prediction of site-specific interim response to chemotherapy.^28^ Punwani et al also showed an inverse correlation between pretreatment ADC(mean) and SUV(max) in 39 patients with proven Hodgkin Lymphoma (Figure 5). Following treatment with two cycles of vincristine, etoposide, prednisolone and doxorubicin (OEPA), median post-treatment ADC(mean) values were significantly higher, and median SUV(max) values significantly lower than pretreatment values.^29^

However, in a study of 50 patients undergoing WBMRI and 18-FDG-PET/CT, both at diagnosis and after 2–3 cycles of chemotherapy, Latifoltojar et al found WBMRI underestimated interim disease response in paediatric and adolescent Hodgkin’s Lymphoma. They found a 74% discordance in staging, and an underestimate of disease response in 26% compared to 18-FDG-PET/CT.^30^

One criticism of using the STIR technique is the inability of STIR sequences to differentiate therapy-induced bone marrow signal abnormalities from lymphomatous involvement.^23^ At our institution, we utilise a WBMRI *two point Dixon technique*. Red marrow is mildly T1 hyperintense relative to skeletal muscle and intervertebral disks owing to its inherent fat content. As a result, areas of red marrow lose signal on out-of phase T1 sequences due to chemical shift artefact. Conversely, areas of disease infiltration appear lower in T1 signal than skeletal muscle and are not subject to the same extent of chemical shift artefact.^32^

The preceding therefore suggests that although WBMRI shows a great deal of promise in paediatric and adolescent Hodgkin’s Lymphoma, for the time-being at least, it cannot replace conventional staging pathways.

#### Langerhans Cell Histiocytosis

**Figure 6.**
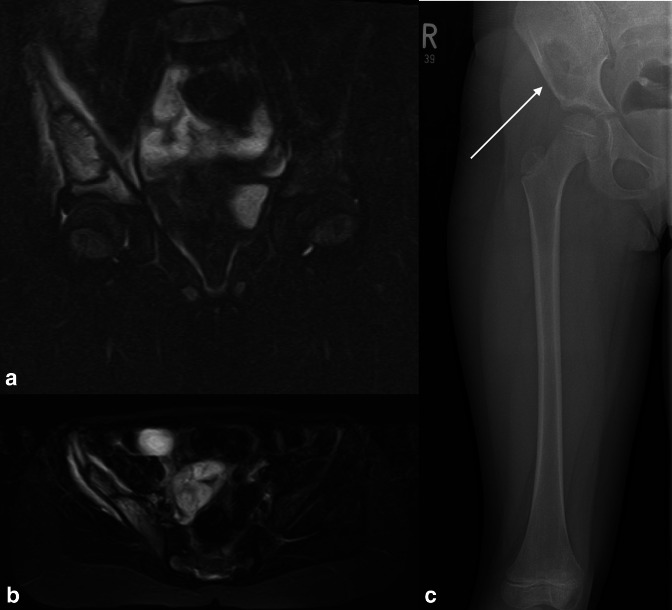
WBMRI in a 6-year-old with langerhans cell histiocytosis. Coronal (**A**) and axial (**B**) STIR MRI shows a focal region of well-defined high T2 signal with a peripheral low signal rim in the right iliac wing. There is bone marrow oedema in the ileum and ischium, and surrounding soft tissues. Plain radiography shows a classic corresponding well-defined lytic abnormality without peripheral sclerosis and a narrow zone of transition (**C**).

The extent of disease in Langerhans Cell Histiocytosis (LCH) is a major predictor of outcome, with multifocality necessitating more prolonged treatment.^33^ The conventional modality for investigating the extent of skeletal disease in LCH is with a radiographic skeletal survey or previously, bone scintigraphy. Investigation of visceral involvement is via an appropriate cross-sectional modality, for example with CT in the context of suspected pulmonary involvement. However, WBMRI has been reported to be useful in detecting skeletal and extra skeletal metastases, and leading to a change in treatment.^34^ Lesions appear as well-defined lytic regions on radiography with corresponding fluid signal on STIR with or without a corresponding soft tissue component and surrounding soft tissue oedema (Figure 6).

Kim et al compared lesion detectability and the accuracy of risk stratification between skeletal survey, bone scan and WBMR in 46 patients with biopsy-proven LCH. Although not all LCH lesions were biopsied, the diagnosis of each suspected LCH lesion was made on the basis of follow-up imaging and consensus between two radiologists. WBMRI using coronal STIR, coronal T1 and post-gadolinium T1 with fat suppression had a far higher sensitivity (99.0%) than skeletal survey (56.6%) and bone scintigraphy (38.4%) for LCH lesions, with no significant differences in the number of false-positives.^35^ The most common region of missed lesions was the pelvic bones on both skeletal survey and bone scintigraphy. MRI detected lesions at all extraskeletal sites.

#### Acute myeloid leukaemia

**Figure 7.**
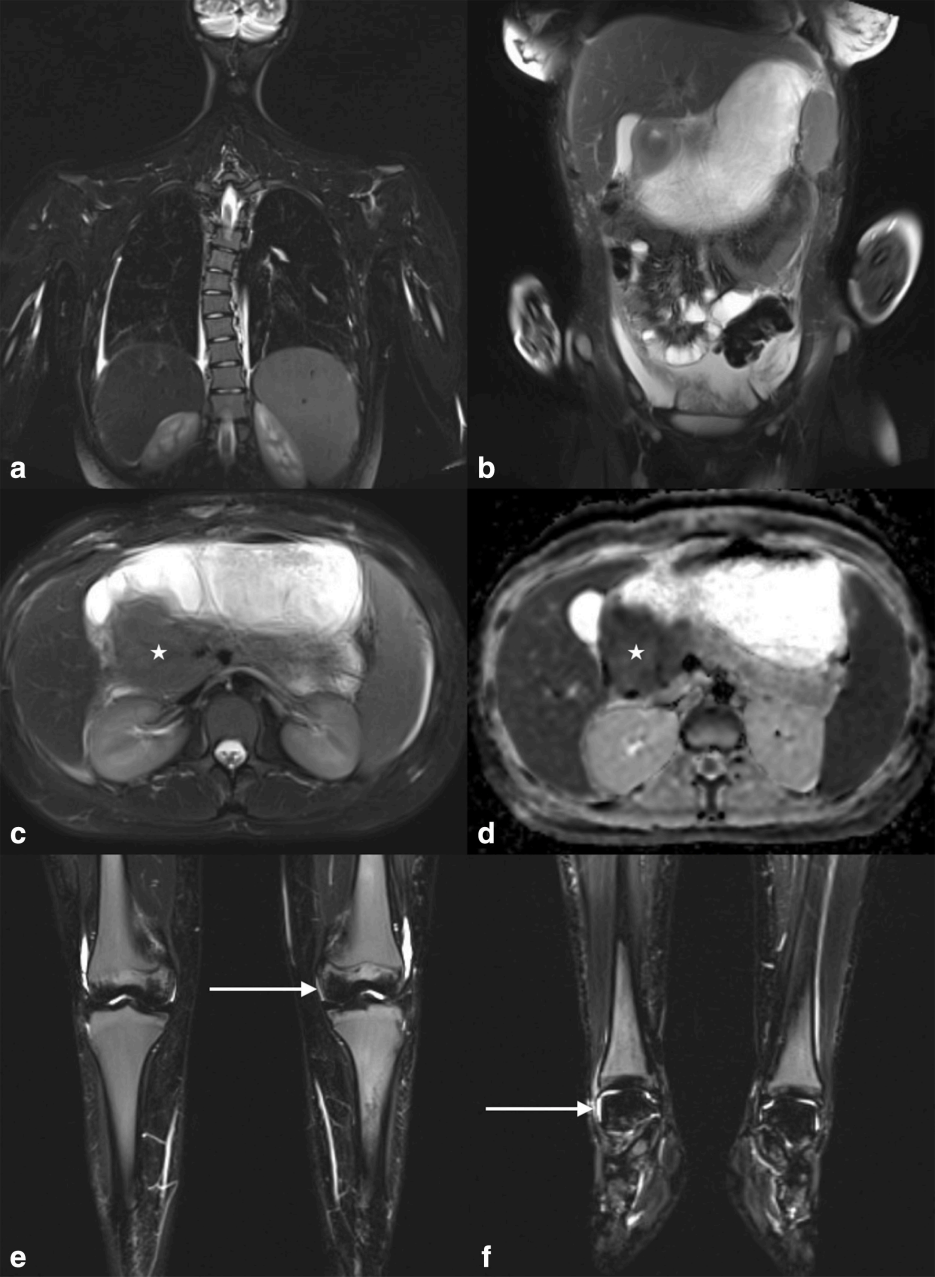
WBMRI of a 14-year-old with acute myeloid leukaemia. Coronal STIR sequences through the chest (**A**) shows a slight thoracic scoliosis, and through the abdomen (**B**) shows a grossly distended stomach. Axial STIR and ADC map show an intermediate STIR, low ADC value right para-aortic mass obstructing the pylorus (stars C and D) in keeping with an eAML tumour. Low STIR signal in the femoral epiphyses and tali (white arrows **E and F**) bilaterally are in keeping with sclerosis secondary to osteonecrosis.

**Figure 8.**
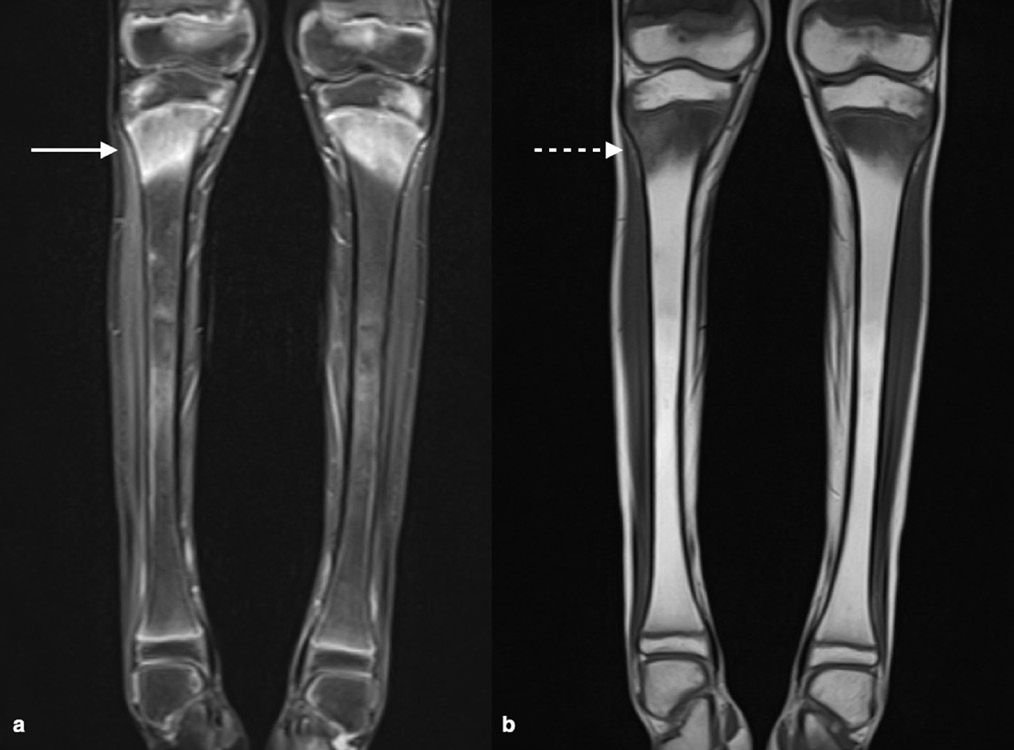
Coronal STIR and *T_1_*-weighted MRI sequences through the lower legs shows well-demarcated metaphyseal high STIR (arrow A) low T1 (dashed arrow B) signal in keeping with leukaemia marrow infiltration in a 9-year-old with acute myeloid leukaemia.

Extramedullary acute myeloid leukemia tumor (eAML), sometimes misleadingly referred to as myeloid sarcoma or chloroma, are rare extramedullary masses comprised of myeloid precursor cells (Figure 7). Although there is no standard recommendation for treatment, isolated eAML tumours may only require local control, for example with radiotherapy. Multifocal disease however, requires systemic chemotherapy. Yoon et al performed WBMRI in 40 patients with AML. They detected a total of 77 eAML tumours, 32 of which were clinically occult in seven patients. This again demonstrates the usefulness of WBMRI in demonstrating clinically non-apparent disease which leads to a change in management^36^ (Figure 8).

### Skeletal metastases

STIR is again the most deployed WBMRI sequence for investigation of skeletal metastases from solid tumours.^37^ Goo et al compared the sensitivity of skeletal metastasis detection of bone scintigraphy, MIBG and CT with WBMRI STIR and found it to be more sensitive than all three ranging between 99 and 100% sensitive^38^.

The specificity of STIR sequences may however be lower than its sensitivity. Goo showed false-positives were high using WBMRI in neuroblastoma, particularly when compared to MIBG in patients with neuroblastoma and ganglioneuroblastoma, demonstrating a positive predictive value of only 7.7%.^38^ Similarly, Krohmer showed 88 false-positives from a population of 130 skeletal and extraskeletal lesions detected by WBMRI in patients with Hodgkin’s Lymphoma, follicular lymphoma, Ewing-sarcoma, Osteosarcoma, fibrosarcoma, rhabdomyosarcoma, alveolar sarcoma and LCH.^39^

However, Daldrup-Link et al showed the sensitivity of WBMRI was a higher than bone scintigraphy, but a lower than 18-FDG-PET, in 51 patients with bone metastases, either proven by histology or follow-up imaging. Primary diseases included Ewing’s sarcoma, osteosarcoma, lymphoma, rhabdomyosarcoma, malignant melanoma and LCH. Despite a lower sensitivity, the false-positive rate of WBMRI was low and maintained a positive-predictive value of 93% compared to 18-FDG-PET^40^ .

### Cancer predisposition syndromes

**Figure 9.**
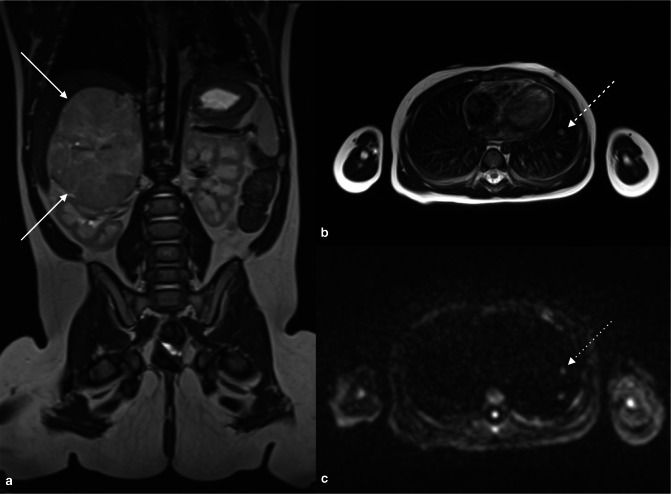
Li Fraumeni syndrome. Coronal T2 SPACE sequence (**A**) in a 9-month-old patient shows a right suprarenal mass confirmed to be an adrenocortical carcinoma at histology. Surveillance Axial T2 (**B**) and DWI (**C**) sequences through the chest at 17 months of age shows two lung nodules in keeping with metastatic recurrence.

Genetic cancer predisposition syndromes (CSP) are those which place children at risk of developing malignancies and include Neurofibromatosis Type 1 (NF-1), Beckwidth-Wiedermann, multiple endocrine neoplasia, Li-Fraumeni and von Hippel Lindau to name a few (Figure 9). Regular screening with WBMRI is attractive in patients with these syndromes owing to the lack of ionising radiation, and theoretical relative risk reduction in developing iatrogenic-induced malignancies.^41–43^ Studies on the efficacy of the technique are sparse however.

Anupindi et al analysed 50 WBMRI examinations of 24 children with paraganglioma-pheochromocytoma syndrome, Li-Fraumeni syndrome and rhabdoid tumor syndrome, using STIR, T1, T2 HASTE and fat-suppressed T2 sequences. Findings were classified as abnormal or incidental, and abnormal findings further classified into high, moderate or low risk for malignancy. Marrow oedema without a discrete mass, and without periostitis were categorised as low risk for malignancy (<20%). Marrow oedema in an unusual location with focal or mass like features, were classified as moderate risk (20–80%). Discrete solid masses, or marrow oedema associated with periosteal reaction, were classified as high risk for malignancy (>80%). Nine findings suspicious for malignancy were identified, including two high-risk, two moderate-risk and five low-risk lesions. Only one high-risk lesion, detected on a coronal STIR sequence, was proven by biopsy to be a papillary thyroid carcinoma. The remainder were benign yielding a useful negative predictive value of 100%, amongst this small population at least.^42^

#### Neurofibromatosis

**Figure 10.**
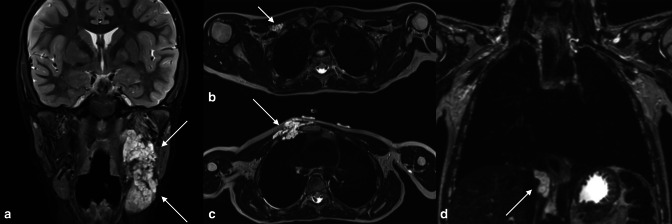
An 8-year-old with neurofibromatosis Type 1. Coronal STIR sequence (**A**) through the face shows a high signal plexiform neurofibroma extending from the left masticator space to below the level of the left mandibular ramus. Further plexiform neurofibromata are shown in the right supraclavicular fossa (**B**), right anterior chest wall (**C**) and at the diaphragmatic hiatus (**D**).

Nguyen et al used whole-body STIR sequences to investigate the frequency of plexiform neurofibomas in 65 children with Neurofibromatosis Type 1 (NF-1).^44^ They detected 73 neurofibromas in 37 children; 18 of which caused clinical symptoms in 17 patients. Symptomatic lesions were more common in the head and neck and larger than those found elsewhere. They therefore suggest that WBMRI might allow early detection and regular monitoring to help estimate growth and potential complications, thereby optimising treatment and management planning (Figure 10).

### Osteonecrosis

Osteonecrosis is increasingly linked to systemic corticosteroids in chemotherapy protocols,^45^ with large juxta-articular lesions at risk for a poorer outcome. Early diagnosis is critical, as the many interventions designed to prevent joint deterioration or delay total joint arthroplasty (such as bisphosphonate therapy), are effective only when initiated prior to detection of substantial joint collapse. WBMRI has been shown to demonstrate clinically occult lesions in children, and those not apparent on plain radiography, thus facilitating early medical and surgical intervention.^46^

**Figure 11.**
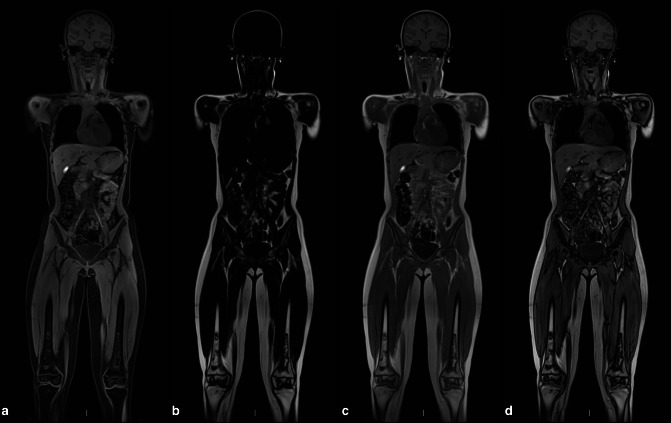
Whole-body coronal T1 Dixon MRI examinations showing water only (**A**), fat only (**B**), in-phase (**C**) and out-of-phase (**D**) sequences. Focal regions of medullary osteonecrosis can be seen in the distal femoral metaphases and epiphyses. The bone marrow otherwise returns normal signal with homogeneous high T1 signal in the fat-only, in-phase and out-of-phase sequences.

Osteonecrosis appears as a well-circumscribed lesion with a low-signal intensity rim against normal high signal marrow on T1 (Figure 11), and high-signal against the attenuated marrow signal on STIR sequences. It is more common in the lower extremities.^47^

Castro compared joint-specific MRI and WBMRI in 40 patients with juvenile systemic lupus erythematous and a history of at least 3 months of steroid use. Osteonecrosis was demonstrated in seven patients on joint-specific MRI, whereas WBMRI using STIR sequences was detected in six.^48^

In a small study, Littooij identified osteonecrosis on WBMRI in 10 of 24 patients after OEPA chemotherapy for Hodgkin’s Lymphoma. Eight patients had osteonecrosis after two cycles of OEPA, with another two developing osteonecrosis after completion of four cycles. Epiphyseal involvement of long bones was seen in 4 of 10 children.^49^

## Conclusion

In 2018, the Oncology Task Force of the ESPR published recommendations for the implementation of whole-body magnetic resonance imaging in paediatric oncology. They acknowledged the potential benefits of WBMRI but given the lack of clinical trials and protocol harmonisation, the publication is a consensus statement by experts in the field, describing the technical prerequisites and protocols to maximise diagnostic confidence. Amongst the recommendations, the group highlighted the sensitivity of STIR sequences in regarding bone marrow changes and the risk of overinterpretation of normal findings as pathology, and that additional imaging might be necessary in particular tumour entities such as the use of tailored MRI of the extremities for skip lesions in osteosarcoma.^1^

Randomised-controlled clinical trials are required for validation, but given their merits outlined above, a combination of STIR, Dixon T1 and DWI, WBMRI could prove both sensitive and specific for investigation of a range of inflammatory and neoplastic diseases in children.
